# XopZ and ORP1C cooperate to regulate the virulence of *Xanthomonas oryzae* pv. *oryzae* on Nipponbare

**DOI:** 10.1080/15592324.2022.2035126

**Published:** 2022-02-19

**Authors:** Hongtao Ji, Taoran Li, Xiaochen Li, Jiangyu Li, Jiayi Yu, Xin Zhang, Delong Liu

**Affiliations:** Institute of Integrative Plant Biology, School of Life Sciences, Jiangsu Normal University, Xuzhou, China

**Keywords:** *Xanthomonas oryzae* pv. *oryzae*, rice, T3SS effector XopZ, oxysterol-binding related protein ORP1C

## Abstract

Bacterial leaf blight caused by *Xanthomonas oryzae* pv. *oryzae* (*Xoo*) has always been considered to be one of the most severe worldwide diseases in rice. *Xoo* strains usually use the highly conserved type III secretion system (T3SS) to deliver virulence effectors into rice cells and further suppress the host’s immunity. Previous studies reported that different *Xanthomonas* outer protein (Xop) effectors include XopZ from one strain appear to share functional redundancies on suppressing rice PAMP-triggered immunity (PTI). But only *xopZ*, except other *xop* genes, could significantly impaire *Xoo* virulence when individually deleting in PXO99 strains. Thus, the XopZ effector should not only suppress rice PTI pathway, but also has other unknown indispensable pathological functions in PXO99–rice interactions. Here, we also found that ∆*xopZ* mutant strains displayed lower virulence on Nipponbare leaves compared with PXO99 strains. We identified an oxysterol-binding related protein, ORP1C, as a XopZ-interacting protein in rice. Further studies found that rice ORP1C preliminarily played a positive role in regulating the resistance to PXO99 strains, and XopZ–ORP1C interactions cooperated to regulate the compatible interactions of PXO99-Nipponbare rice. The reactive oxygen species (ROS) burst and PTI marker gene expression data indicated that ORP1C were not directly relevant to the PTI pathway in rice. The deeper mechanisms underlying XopZ–ORP1C interaction and how XopZ and ORP1C cooperate for regulating the PXO99–rice interactions require further exploration.

To defend against invasion by numerous potential pathogenic microbes, plants have gradually evolved a complex, multilayered immune system. Plant immune systems usually include two defensive layers.^1^ In the first layer, plants recognize the pathogen-associated molecular patterns (PAMPs) of invasive pathogens through pattern recognition receptors (PRRs) that mediate basal PAMP-triggered immunity (PTI). PTI responses usually include reactive oxygen species (ROS) burst, mitogen-activated protein (MAP) kinases activation, callose deposition, and PTI marker genes expression.^2,3^ In the second layer, plants have achieved the ability to recognize attacks of numerous virulence effectors through resistance (R) proteins that activated effector-triggered immunity (ETI).^1,4^ ETI is usually associated with the hypersensitive response (HR), a rapid and restricted cell death at infection sites.^5^ The co-evolution between the virulence effectors of pathogens and R proteins of plants together led to the so-called “zigzag model”.^1^ Traditionally, the PTI and ETI pathways are separated, but recent studies have suggested that ETI signaling depends on PTI, PTI and ETI cooperate to regulate plant immunity.^6,7^

To infect a host, pathogenic bacteria usually translocate effectors into the host cell through a type III secretion system (T3SS) and further suppress the plant innate immunity. T3SS effectors usually act kinds of biochemical roles, including protein kinases, phosphatases, proteases, acetyltransferases, and E3-ubiquitin ligases, among others.^8,9^ Mechanistically, T3SS effectors have been found to interfere with various different host defense pathways, usually including MAPK signaling, phytohormone signaling, cytoskeleton formation, proteasome- or autophagy-dependent protein degradation, and host gene expression, among others.^8,9^ To understand the biological functions of T3SS effectors in host cells, it is key to identify plant targets and further explore the mechanism of effector-triggered modifications in different plant cellular processes.

Bacterial leaf blight caused by *Xanthomonas oryzae* pv. *oryzae* (*Xoo*) has always been considered to be one of the most severe worldwide diseases in rice.^10^ Similar to many other gram-negative pathogenic bacteria, *Xoo* strains also use the highly conserved T3SS to deliver numerous T3SS effectors into rice cells.^11^ However, unlike with other pathogenic bacteria, *Xanthomonas* T3SS effectors are categorized into two groups: transcription activator-like (TAL) effectors and *Xanthomonas* outer protein (Xop) effectors, which are also called non-TAL effectors.^12,13^ Most TAL effectors have been identified as eukaryotic transcription activators and further transcriptionally activate the corresponding target genes for host disease resistance or susceptibility.^14,15^ For example, PXO99 ∆*pthXo1* mutant strains have completely lost their virulence toward Nipponbare and other susceptible rice cultivars. Further studies found that the TAL effector PthXo1 could specifically bind the promoter region of the *OsSWEET11* gene and up-regulate its expression in rice cells. The SWEET protein coded by the *OsSWEET11* gene belongs to the membrane sugar transporter family, which provides nutrients for pathogenic bacteria growth through transporting the intracellular sugar to the apoplasts.^16^ Tian et al. identified an *Xoo* resistance gene *Xa10* which stimulated by the TAL effector AvrXa10 in IRBB10 rice. Mechanistically, Xa10 is located on the endoplasmic reticulum membrane and induces programmed cell death (PCD) by destroying the intracellular Ca^2+^ balance, and further limits the expansion of *Xoo* lesions.^17^

To date, 22, 20, and 16 Xop effectors have been identified in different *Xoo* strains PXO99, MAFF311018 and KACC10311, respectively. The sequence of Xop effectors in different *Xoo* strains is highly conservative (http://www.xanthomonas.org/t3e.html). Unlike the definite roles that TAL effectors play in *Xoo*-rice interactions, the pathogenic functions of numerous Xop effectors remain unclear or redundant.^18^ Recently, we reported that the XopI effector of the African *Xoo* BAI3 strain could strongly suppress SAR immunity through impersonating and hijacking the host ubiquitin proteasome system (UPS) in rice.^19^ Additionally, other studies found that several Xops have recently been identified to target and suppress the host PTI. XopR from the *Xoo* strains MAFF311018 or PXO99 has been shown to target the receptor-like kinase BIK1 and further block rice PTI.^20,21^ XopAA, XopP, and XopY from the *Xoo* MAFF311018 strain suppress rice PTI through interacting with the host PRR receptor OsBAK1, E3 ubiquitin-ligase OsPUB44, and receptor-like kinase OsRLCK185, respectively.^22–24^ In addition, the XopN, XopQ, XopV, XopX, and XopZ effectors have all been proven to suppress rice PTI pathway, suggesting that numerous Xops effectors from one strain appear to share functional redundancies in suppressing rice PTI, but their specific targets on host PTI pathway are diverse and unknown.^18,25–27^ Interestingly, Song and Yang found that only *xopZ*, except other *xop* genes, could significantly impair *Xoo* virulence on the leaves of IR24 rice when deleted in PXO99 strain.^18^ In summary, we presume that other Xop effectors could also complete the suppression for host PTI in PXO99∆*xopZ*-rice interactions, XopZ effector should not only suppress host PTI, but also has other unknown indispensable pathological functions in PXO99-rice interactions.

Two equivalent *xopZ* effector genes (PXO_01041 and PXO_06125) were identified in PXO99 genome due to a 212-kb sequence duplication.^18,28^ In a previous study, Song and Yang constructed the PXO99 mutants ∆*xopZ* strain by using traditional homologous recombination methods that introduced kanamycin resistance cassettes at two *xopZ* sites in PXO99 genome.^18^ However, in this study, we constructed the PXO99 mutants ∆*xopZ* (both PXO_01041 and PXO_06125 deletions) by using a marker-free deletion method. We then evaluated the virulence of *xopZ*-related strains on Nipponbare rice leaves. The inoculation data showed that the *xopZ* deletion significantly impaired the *Xoo v*irulence, the average lesion lengths on Nipponbare leaves inoculated with ∆*xopZ* and ∆*xopZ*/pHMI strains were significantly shorter than those on rice leaves inoculated with PXO99 and ∆*xopZ*/*xopZ* strains (Figure 1a,b), and the populations of ∆*xopZ* and ∆*xopZ*/pHMI strains in Nipponbare leaves were substantially lower than PXO99 and ∆*xopZ*/*xopZ* strains (Figure 1c). In summary, consistent with the important role of the XopZ effector played in the PXO99-IR24 rice,^18^ our experiments also indicated that XopZ is indispensable for compatible interaction of PXO99-Nipponbare rice (Figure 1). However, when the same mutant strain PXO99∆*xopZ* was inoculated on Kitaake rice leaves, no obvious virulence reduction was observed.^27^ This might be due to the genetic background of the host rice or the specialized interaction of host-pathogen race.

**Figure 1.**
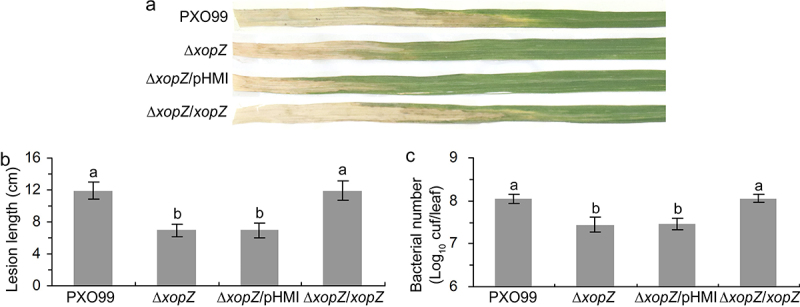
XopZ effector contributes to virulence of PXO99 strain on Nipponbare. (a) Pathogenic phenotypes of *xop*Z-related strains on Nipponbare rice leaves photographed at 14 days post-inoculation (dpi). (b) Length measurement of the blight lesions at 14 dpi. Each bar represents the mean and standard deviation (SD) of 10 rice leaves. (c) Statistics of *Xoo* populations in Nipponbare leaves at 14 dpi. Each bar represents the mean and SD of 5 rice leaves. In (b) and (c), different letters indicate significant differences based on Duncan’s multiple range analysis (P < 0.05). These experiments were repeated three times and similar results were obtained.

To date, the specific host targets of XopZ have not been characterized. We further screened for rice proteins that interact with XopZ by yeast two-hybrid (Y2H) assay. First, yeast strains transformed with bait plasmids and Nipponbare cDNA libraries were mixed for conjugation growth on synthetic dextrose (SD) medium, and ORP1C (accession number: LOC_Os03g49770) was identified as a candidate target to interact with XopZ according to sequencing of numerous positive clones. ORP1C belongs to the oxysterol-binding related protein family (ORPs).^29^ The interaction between XopZ and the full-length ORP1C protein was further confirmed according to the phenotype of yeast growth phenotype and X-gal visualization of the paired Y2H assay (Figure 2a). Next, we explored whether XopZ could bind to ORP1C in rice cells. The XopZ-HA plasmid was transiently expressed together with ORP1C-Flag in Nipponbare rice protoplasts, and subsequent co-immunoprecipitation (Co-IP) tests revealed that XopZ could specifically bind to ORP1C in rice cells (Figure 2b).

**Figure 2.**
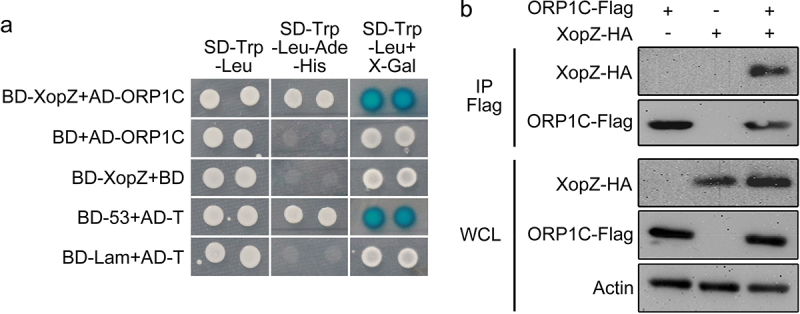
XopZ specifically bind to ORP1C in yeast and rice cells. (a) Y2H assay identifying the interaction between XopZ and ORP1C in yeast cells. The transformant expressing BD-53 and AD-T was used as a positive control. The transformants expressing BD-Lam and AD-T, BD-XopZ and empty AD, empty BD and AD-ORP1C were used as negative controls. (b) Co-IP assays identifying the interaction between XopZ and ORP1C in rice protoplasts. Whole-cell lysates (WCL) of rice protoplasts transformed with different plasmid combinations were prepared and analyzed by immunoblotting with marked antibodies, Co-IP was conducted with the Flag antibody (IP Flag) and the interaction was further analyzed by immunoblotting with Flag or HA antibodies.

To explore the biological functions of *ORP1C* in rice cells, we cultivated *ORP1C* gene mutant rice with the CRISPR-Cas9 gene editing system. First, we calculated and constructed the specific sgRNA to target the nucleotides of the *ORP1C* gene from 283 to 305 (Figure 3a). The plasmids containing the specific sgRNA and Cas9 expression cassette were introduced into Nipponbare rice calluses. In total, two homozygous mutant rice lines (*ORP1C*#2 and *ORP1C*#9) were identified according to the sequencing results in transgenic progenies. *ORP1C*#2 had a homozygous allelic mutation with 2-bp deletion, *ORP1C*#9 had a 1-bp insertion in the target gene sites (Figure 3b). These two deletions or insertions both caused frameshift mutations and early stop codons.

**Figure 3.**
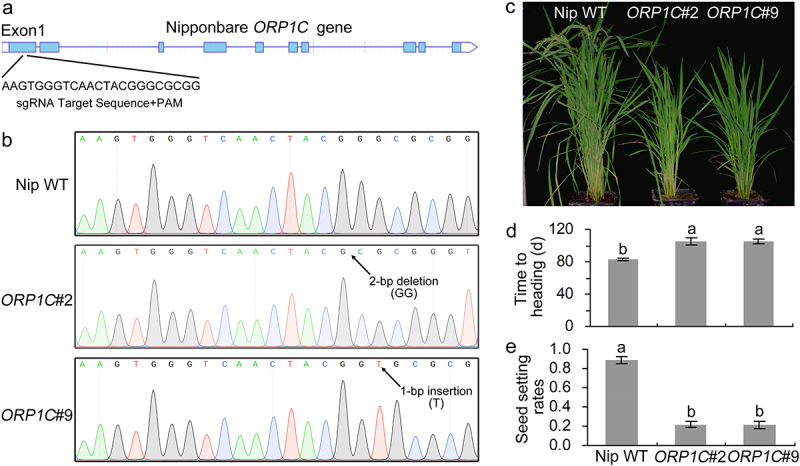
Identification of *ORP1C* mutant rice generated by the CRISPR-Cas9 gene editing system. (a) Calculation of the sgRNA target sites based on *ORP1C* sequence analysis. (b) Sequencing identification of *ORP1C* mutant rice lines. (c) Growth phenotypes of 100-day-old Nipponbare WT and *ORP1C* mutant rice. (d) Days to heading of different rice lines. (e) Seed setting rates of different rice lines. The seed setting rate was calculated as the ratio of fully filled seeds to the total number of grains. In (d) and (e), each bar represents the mean and SD of 5 rice plants. Different letters indicate significant differences based on Duncan’s multiple range analysis (P < 0.05). These experiments were repeated three times with similar results obtained.

Most eukaryotic genomes encode oxysterol-binding proteins (OSBPs) and OSBP-related proteins (ORPs). These OSBPs/ORPs have been widely studied in humans and fungi,^30,31^ and are indispensable for diverse cellular functions, including signaling cascades, endosomal movements, membrane trafficking, tumor metastasis, cell cycle progression, and so on.^31^ Mechanistically, OSBPs/ORPs act as a classical non-vesicular lipid transporter that sense and transfer kinds of lipids, especially kinds of sterols and phospholipids, and further regulate the lipid distribution at the different cell membranes.^31^ However, the unique structural basis of numerous OSBPs/ORPs for sensing and transferring different kinds of specific lipids are not fully understood.^32,33^ Currently, 12 and 6 OSBP/ORP genes have been identified in the *Arabidopsis* and rice genomes, respectively.^29^ The TAIR database demonstrates that different OSBP/ORP genes are expressed at different levels under numerous stress conditions in *Arabidopsis*.^29^ However, the definite physiological and developmental functions of these OSBPs/ORPs in plants remain unknown. In particular, whether or how OSBPs/ORPs are involved in regulating lipids trafficking remains to be determined in plants.

During this study, we observed some significant changes in the morphological and agronomic phenotypes in the *ORP1C* mutant rice as compared to the Nipponbare WT rice under identical natural conditions. The *ORP1C* mutant rice exhibited a slight dwarf and late-heading phenotype in the adult stages (Figure 3c,d). In addition, *ORP1C* mutant rice also exhibited a mild sterile phenotype in the harvest period, and the seed setting rates of the *ORP1C* mutant rice were all highly lower compared with Nipponbare WT rice (Figure 3e). These data indicate that the *ORP1C* may play an important role in regulating the growth and yield of rice.

We further explored the biological functions of the *ORP1C* gene in PXO99–rice interactions. The statistical data show that the average lesion lengths on the leaves of the *ORP1C*#2 and #9 mutants were apparently longer than those on the leaves of WT rice (Figure 4a,b). Above results preliminarily indicate that ORP1C positively regulates the resistance to PXO99 strains in rice. In addition, according to the statistical data of lesion length, we found that *∆xopZ* strains displayed impaired virulence on Nipponbare WT leaves compared with PXO99 or *∆xopZ*/*xopZ* strains, but these three strains (PXO99, ∆*xopZ* and ∆*xopZ*/*xopZ*) displayed a nearly equivalent severe virulence on *ORP1C* mutant rice (*ORP1C*#2 and *ORP1C*#9) (Figure 4a,b). These results preliminarily suggest that the virulence function of XopZ is dependent on ORP1C, ORP1C should be the specific host targets of the XopZ effector and further cooperate to regulate the compatible interaction of PXO99-Nipponbare rice.

**Figure 4.**
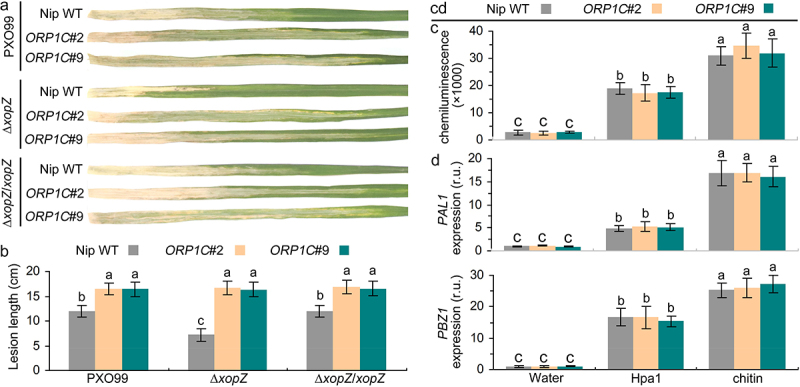
XopZ and ORP1C cooperate to regulate the virulence of PXO99 strain on Nipponbare not through the host PTI pathway. (a) Pathogenic phenotypes of *xop*Z-related strains on different rice leaves photographed at 14 dpi. (b) Length measurements of blight lesion at 14 dpi. Each bar represents the mean and SD of 10 rice leaves. These experiments were repeated three times with similar results obtained. (c) Measurement of ROS bursts in rice leaves after treatment with different elicitors. (d) Expression analyses of *PAL1* and *PBZ1* gene in different rice leaves after treatment with different elicitors. The average expression levels of *PAL1* and *PBZ1* genes in Nipponbare WT leaves treated with control water solution are defined as “1”. In (c) and (d), each bar represents the mean and SD of three biological replicates, five independent experiments were repeated with similar results obtained. In (b) to (d), different letters indicate significant differences based on Duncan’s multiple range analysis (P < 0.05).

For the benefit of survival and proliferation, recent advances have revealed that numerous intracellular and cell-associated animal pathogenic bacteria have evolved a series of efficient strategies to gain advantages by hijacking host lipids metabolism.^34–38^ However, to date, relevant studies on the hijacking of lipid metabolism by plant pathogenic bacteria remain scarce.^39,40^ In this study, we have found that the XopZ effector of PXO99 could specifically bind to the rice OSBP-related protein ORP1C (Figure 2), and interfere with the resistance function of ORP1C in PXO99–rice interactions (Figure 4a,b). However, the mechanisms underlying how ORP1C positively regulates the resistance of rice to PXO99 remain unknown.

In previous studies, XopZ was proved to suppress the host basal PTI defense in *Nicotiana benthamiana* leaves or rice protoplasts,^18,27^ however, this PTI suppression effect of XopZ have not been proven to have a direct relationship with the virulent role of XopZ played in compatible interaction of PXO99-rice.^18^ Hpa1 proteins from *Xoo* strains have been recognized as PAMPs and further activate the PTI response on different plants, similar to other PAMPs.^41^ Herein, we found that the Hpa1 protein and fungal classical PAMP chitin could both activate the rice PTI response by inducing a ROS burst (Figure 4c) and up-regulating the expression of PTI marker *PAL1* and *PBZ1* genes in different rice leaves (Nipponbare WT, *ORP1C*#2, or #9 mutants leaves), equivalently (Figure 4d). These data preliminarily indicate that ORP1C plays no role in regulating the rice PTI pathway, and XopZ–ORP1C interaction cooperates to regulate the compatible interaction of PXO99-Nipponbare rice not through the rice PTI pathway. In summary, the deeper mechanisms underlying XopZ–ORP1C interactions and how XopZ and ORP1C cooperate for regulating the PXO99–rice interaction require further exploration.

## Materials and methods

### Plant materials

Nipponbare WT or *ORP1C* mutant rice were used, and were grown in growth chambers, greenhouses, or fields as described previously.^19^ For seed breeding and determination of the heading time, rice plants were grown in Xuzhou City of Jiangsu Province in China under natural filed conditions from June to October. The *ORP1C* mutant rice lines were generated with the CRISPR/Cas9 gene editing system.^19^ Briefly, the oligonucleotides targeting the *ORP1C* gene were calculated on Cas-Designer website (http://www.rgenome.net/cas-designer/). The synthesized *ORP1C* oligonucleotides were linked into the plant CRISPR/Cas9 plasmids pRGEB32, and this pRGEB32-*ORP1C* plasmid was further introduced into Nipponbare calli by an *Agrobacterium*-mediated transformation assay as previous methods.^42^ For mutant identification, the target and flanking *ORP1C* gene fragments amplified from rice genomic DNA were further sequenced using an corresponding primer.

### Microbial strains

Different microbial strains including *Escherichia coli, Agrobacterium tumefacien, Yeast* and *Xoo* strains were used and cultured as described previously.^19^ Two equivalent *xopZ* effector genes (PXO_01041 and PXO_06125) were found in the PXO99 genome due to a 212-kb sequence duplication,^18,28^ the *xopZ* genes (both PXO_01041 and PXO_06152) were both deleted following an marker-free deletion method with two cycles by the pK18mobsacB plasmid as described previously,^19^ and the ∆*xopZ* (both PXO_01041 and PXO_06125 deletions) mutants were screened with sucrose-positive and kanamycin-negative phenotypes on nutrient agar (NA) plates, and eventually identified by the PCR assay. The ∆*xopZ*/*xopZ* complementation and ∆*xopZ*/pHM1 control strains were also constructed with the assistance of the pHM1 plasmid as described previously.^19^

### Xoo *virulence assay*

*XopZ*-related strains were cultured in nutrient broth (NB) medium overnight. Inoculated strains were resuspended in dH_2_O at OD_600_ = 0.6, and inoculated on 45-day-old rice by the leaf clipping assay.^19^ The pathogenic phenotypes of different *Xoo* strains were identified by photographing or surveying the leaf blight lesion at 14 dpi. The populations of different *xopZ*-related strains in different rice leaves were calculated as described previously.^19^

### Protein interaction assays

The Y2H experiments were performed following previous studies.^19^ The *xopZ* gene was linked into the bait plasmids pGBKT7. The yeasts transformed with bait plasmids were then co-cultured with yeasts-transformed cDNA libraries of Nipponbare rice leaves (GeneCreate Biotech). Conjugated yeasts were screened on SD-WL (without Trp and Leu) and SD-WLAH (without Trp, Leu, Ade, and His) plates. Rice library plasmids from positive yeast cells of SD-WLAH were introduced into *E. coli* and further sequenced. The full-length XopZ-ORP1C protein interactions were identified by yeast growth on SD-WLAH plates that were co-transformed with pGBKT7-*xopZ* and pGADT7-*ORP1C*. X-Gal agarose mixtures for detecting the lacZ activity were overlaid on yeast SD-WL plates to further confirm their interactions.

For the Co-IP assay, the plant transient expression plasmids pUC35s-XopZ-HA and pUC35s-ORP1C-Flag were transiently expressed in Nipponbare rice protoplasts alone or together. Whole-cell lysates (WCL) of rice protoplasts were prepared and analyzed by immunoblotting with Flag, HA or Actin antibodies (Beyotime) at 12 h after transient expression. Meanwhile, WCL was incubated with Flag antibody for 16 h at 4°C. The mixture was then incubated with Dynabeads protein A (Beyotime) for 5 h at 4°C. Immunoprecipitated proteins were separated in 8% SDS-PAGE gels and immunoblotted by Flag or HA antibodies.^19^

### ROS burst measurement

The ROS bursts were measured with the luminal-dependent chemiluminescence assay.^43^ Leaf disks from 21-day-old rice were collected and incubated in dH_2_O for 12 h. Five leaf disks per sample were placed in a microcentrifuge tube containing 100 uL of luminol (Bio-Rad), 1 uL of horseradish peroxidase (Beyotime), and elicitors (50 nM chitin, 5 μg/mL Hpa1 protein solutions or water as control) to test the ROS bursts. The luminescence was measured using Varioskan LUX (Thermo Scientific) at 1 h after treatment.

### Gene expression analysis

To analyze the expression levels of *PAL1* and *PBZ1* genes, rice leaves treated with the Hpa1 protein or chitin solutions were collected and ground together at 9 h. Rice RNA was extracted with an RNAprep pure Plant Kit (Tiangen), and reverse transcribed into cDNA with HiScript II Reverse Transcriptase (Vazyme). qRT-PCR was conducted using the ChamQ SYBR qPCR Master Mix (Vazyme) on an StepOnePlus Real-Time PCR system (Applied Biosystems). The constitutively expressed *Ubi1* (rice) genes were selected as references.
